# The Practice of Pre-trial Detention in England & Wales - Changing Law and Changing Culture

**DOI:** 10.1007/s10610-022-09504-y

**Published:** 2022-02-16

**Authors:** Tom Smith

**Affiliations:** University of the West of England, Bristol, UK

**Keywords:** Pre-trial detention, Remand, Bail, Magistrates, Disclosure, Fieldwork

## Abstract

Pre-trial detention empowers criminal courts to imprison defendants before they have been convicted of an offence. This is a significant power which should be subject to a rigourous decision-making process. A 2016 study of pre-trial detention practice in England and Wales highlighted concerns about such processes, recommending changes to law and practice in that jurisdiction. In 2017, several of these recommendations became law. This article details a follow-up empirical study, conducted in 2020, which sought to examine the impact of these changes on day-to-day pre-trial detention practice in criminal courts. After analysing the data, the article concludes that the changes in fact had minimal impact on practice, and suggests that changing the law does not necessarily translate into a change in the culture of pre-trial detention practice.

## Introduction

The use of pre-trial detention by criminal courts (hereafter, PTD) inherently inhibits the right to liberty and implicitly challenges the presumption of innocence. This invasive power, deemed necessary in various circumstances, affects a significant number of unconvicted citizens every year. England and Wales (hereafter, E&W) has one of the lower global proportions of defendants detained prior to trial (hereafter, pre-trial detainees), and has been cited as a comparative leader in its responsible use. However, after a period of decline and stability, recent years have seen the gross number of pre-trial detainees rising in this jurisdiction. Furthermore, a number of concerns have been identified in previous research on PTD in E&W, suggesting that aspects of the current law and practice in the jurisdiction risk driving overuse of detention by courts. This article seeks to examine whether such concerns have been effectively addressed. This will be achieved by providing an overview of the law governing PTD in E&W; reviewing a significant piece of research conducted in 2016 by Cape & Smith, identifying some of the key issues highlighted; examining the legal and regulatory changes that occurred as a result of this; and finally reporting on recent fieldwork designed to measure the impact of these changes on the practice of PTD in E&W courts. The article concludes by reviewing whether changes in the law resulted in changes in practice, and finally considering the feasibility of achieving changes to practice through research and legal reform.

## The Law Governing Pre-trial Detention in England and Wales

This section provides a brief overview of the fundamental aspects of PTD law in E&W – for a fuller explanation, see Smith (2021). Since this article seeks to examine the impact of a change in the law in this area, it is also important to note that the current legal framework is different to that which existed at the time of the original research conducted by Cape & Smith. In short, pre-2016, the law was identified as deficient in several ways; the legal framework was changed as a result of the research; and now reflects the legal framework discussed in this article. The use of detention and bail by courts – generally referred to as remand in E&W – is primarily governed by the Bail Act (BA) 1976, and various other statutes and sub-statutory regulations (most notably, the Criminal Procedure Rules (CrimPR), 2020). In E&W, criminal trials are dealt with by Magistrates’ Courts or the Crown Court (the lower and higher trial courts respectively). Any hearing which takes place prior to trial or sentence must conclude with a decision about PTD – in short, whether a defendant can be released or detained until the next hearing (Magistrates Court Act, 1980; Senior Courts Act, 1981; Crime and Disorder Act, 1998). The BA 1976 enshrines one of the central principles of PTD regulation in E&W – the rebuttable presumption in favour of a defendant’s release on bail (under Section 4 (1)). The default position is that a defendant must be released on bail (on the condition that they surrender to custody on a specified future date); but this can be overturned if one or more statutory exceptions to bail are satisfied. To detain a defendant, the court must be satisfied that there are ‘substantial’ grounds for doing so – broadly, based on the risk a defendant will fail to surrender; interfere with evidence or witnesses; or commit further offences – with the criteria for doing so varying depending on the alleged offence.

The legislation requires decision makers to engage in a process of examining the features of each individual case to establish whether detention is justifiable. This implies that a thoughtful, individualised rationale is expected for each PTD decision. The courts can attach non-custodial requirements to any release on bail, falling into one of two categories: what can be broadly described as ‘money’ bail (used rarely in E&W), and the more general and flexible ‘conditions’. ‘Conditions’ – described in the legislation as ‘such requirements as appear to the court necessary’ – are theoretically unlimited in scope, providing the courts with a range of tools for regulating the behaviour of released defendants when detention is not appropriate (BA, 1976). Conditions – such as a curfew, a requirement to regularly report to a police station, or electronic monitoring – are justified as necessary on the same grounds as detention (risk of failing to surrender, interference, and/or further offences). A defendant must either attend (in person or otherwise) at a PTD hearing or have the opportunity to make representations (CrimPR, 2020), normally through a duty lawyer or legally-aided representative.^1^ The CrimPR 2020 require Initial Disclosure of the Prosecution Case (hereafter, IDPC) and provision of any information (to both the court and a defendant) which is relevant to PTD decision-making. The extent and timing of the information provided to all the parties, including the defence, is important in making an appropriate remand decision.

The actual use of PTD by criminal courts in E&W has varied over the last two decades. The number of pre-trial detainees fluctuates from day to day due to the constant flow of departures from, and arrivals into, pre-trial custody. The population remained relatively stable in the mid-2010 s, with approximately 10,000 persons in PTD on any given day between 2014 and 2019 (Ministry of Justice and HM Prison Service, 2020). This followed a decline in gross numbers of pre-trial detainees from a peak in 2010 (approximately 13,000 persons on any given day). However, the last two years have seen a reversal of this trend of decline and then plateau, with numbers once again rising to a peak of 12,727 in June 2021 (Ministry of Justice and HM Prison Service, 2021). A common yardstick for comparing the use of PTD across jurisdictions is the proportion of the prison population which is untried or unsentenced. As of June 2021, this amounted to 16% of prisoners in E&W (Ministry of Justice and HM Prison Service, 2021). Whilst this is one of the lower global proportions (Aebi & Tiago, 2021; World Prison Brief and Institute for Criminal Policy Research, 2021), this figure has limited usefulness on its own. E&W also has a high *per capita* prison population at 131 per 100,000 citizens: one of the highest in Europe and higher than many other developed nations (Aebi & Tiago, 2021; World Prison Brief and Institute for Criminal Policy Research, 2021).^2^ Therefore, the gross number of defendants remanded in custody by courts in E&W (just over 75,000 in the year up to March 2021 (Ministry of Justice, 2021)) is significant: even more so when one considers that the prison remand population has remained stable and then risen whilst the number of cases passing through the criminal courts has been steadily decreasing for the last decade (with a dramatic decrease during the early stages of the Covid-19 pandemic).

## Key Findings of the 2016 Research

In 2016, Cape & Smith published the findings of the first major study of PTD decision-making in E&W courts in many years (see Smith, 2021 for an overview of research in this area prior to 2016). This was part of a cross-jurisdictional project co-ordinated by Fair Trials, and funded by the European Commission (Fair Trials, 2016; Cape & Smith, 2016). The project, which covered 10 European Union member states including E&W, sought to understand the over-use of PTD in many jurisdictions and to inform legal reforms. The study adopted an empirical, mixed methods approach, including a desk-based review of relevant law, procedure and research; a survey of criminal defence practitioners; observation of PTD hearings; review of prosecution case files; and interviews with practitioners (Cape & Smith, 2016). Among various issues identified, the research found that PTD proceedings were generally very short, averaging only a few minutes, and noted that in many cases more time was dedicated to case management than to PTD decision-making (Cape & Smith, 2016). Additionally, and in line with previous studies (Cape et al., 2010; Cape & Namoradze, 2012), it was found that the reasoning provided for decisions to remand a defendant in custody were often limited, especially in those cases dealt with by Lay Magistrates (hereafter, LMs),^3^ with domestic and human rights ‘routinely breached’ (Cape & Smith, 2016). The research also suggested that judicial decisions were characterised by a lack of engagement with the specific features of individual cases, and identified significant problems with the disclosure of relevant evidence by the prosecution (Cape & Smith, 2016). Evidence provided to the defence was often tardy, minimal or incomplete (Cape & Smith, 2016). As a result, the final report made various recommendations for reform, including that more time and resources should be made available in Magistrates’ Courts for PTD hearings; a clear obligation on the police or Crown Prosecution Service (hereafter, CPS) to provide timely access for the defence to all relevant case materials;^4^ and that the CrimPR should be amended to make clear that the court must devote sufficient time to decision-making and explain its decision by reference to the facts of the case (Cape & Smith, 2016).

## Engagement with Policy Makers and Results of this Process

In March 2016, the research and recommendations of Cape & Smith were shared with the Criminal Procedure Rule Committee (hereafter, the Committee), the body responsible for producing the CrimPR. The Secretary of the Committee (hereafter, the Secretary) distributed a summary of the research findings and relevant excerpts for consideration at a Committee meeting that month. This was followed by the submission of a position paper to the Committee, summarising relevant research findings and outlining recommendations. The paper argued that there was a need to amend the CrimPR because the defence may not receive adequate information at a sufficiently early stage, and that limited time was spent by courts in assessing PTD. The published study and position paper were explicitly considered with a view to possible amendment of the CrimPR at Committee meetings in July and October 2016, and remained pertinent to discussions at a further meeting in November 2016. The Committee concluded that amendments would be introduced which explicitly required the court to take sufficient time to consider PTD; to explain its decision adequately; and required prosecutors to ensure the availability of information relevant to PTD. These were incorporated into the CrimPR via statutory instrument in February 2017, and came into force in April 2017 (with the contribution of the research to the rule changes being acknowledged in an Explanatory Memorandum to the rule changes (Ministry of Justice, 2017)). As a result of this process, the CrimPR thereafter required that courts:

1. ensure that ‘sufficient time’ is taken for PTD decision-making (Rule 14.2(1)(d)(ii));
2. that, where the prosecution wishes to introduce evidence or information that forms part of the Initial Details of the Prosecution Case (IDPC) that has not previously been disclosed, that the defence be given ‘sufficient time’ to consider it (Rules 8.4(2) and 14.2(1)(d)(i));
3. that prosecutors share all relevant evidence and case materials with the defence (and the court) before hearings and in a timely manner (Rule 14.5(2)); and.
4. that courts fully explain the reasoning for their PTD decisions by reference to the specifics of a case (Rule 14.2(5)).

As such, these amendments constituted a substantial procedural step towards improving the rigour of criminal courts in considering PTD, better aligning the law in E&W with European Union and international legal standards, and reflecting the core recommendations of the research by Cape & Smith. Hereafter, these four changes will be collectively referred to as ‘the amendments’ for the purposes of the fieldwork analysis.

## Searching for Impact: The 2020 Study

### Objective

Notwithstanding this significant and positive outcome, changes to the law do not necessarily translate into changes in practice. In short, the “coal face” impact of the amendments was unclear. For example, it was unknown what level of awareness lay magistrates (who are responsible for the vast majority of PTD decisions) had of the changes to the CrimPR in this context. If they had at least some level of awareness, it was unclear whether the Rules as amended were being adhered to and, if they were, to what extent. Without further examination, there existed no direct evidence as to whether the amendments had achieved their purposes: for example, whether sufficient time was being spent on PTD decision-making in court (often meaning more time, in light of the general brevity of PTD hearings); or whether other useful metrics for measuring the rigour of PTD hearings – such as the level of detail in reasoning for decisions or the extent of engagement between the prosecution and defence during hearings – had been positively impacted by the amendments. To this end, the author undertook a follow-up study to examine these metrics, thereby assessing the impact of the amendments.

### Methodology

The follow-up study adopted a mixed methods approach, combining qualitative and quantitative elements via an online survey and court observations. The survey (conducted between March and May 2020)^5^ targeted criminal justice practitioners involved in PTD processes subject to the CrimPR 2020: that is, LMs; District/Deputy District Judges (hereafter, DJs/DDJs); and Defence Practitioners (hereafter, DPs) in Magistrates’ Courts.^6^ The survey consisted of a set of closed/quantitative and open/qualitative questions, specifically examining the impact of the amendments on PTD practice. The survey asked respondents about their awareness of the amendments, and whether they had noticed subsequent changes in practice in PTD decision-making. Questions specifically asked whether time spent on PTD decision-making was, in their view, ‘sufficient’; whether practice in relation to disclosure prior to PTD hearings had, in their view, changed since the amendments; whether defendants and their representatives generally had, in their view, ‘sufficient time’ to consider evidence/information for the purposes of PTD hearings; and whether they had ever acted to ensure such time is available. All of these questions were designed to reflect the amendments. A total of 33 people responded to the survey, all of whom were LMs or DPs.^7^ Not all respondents answered all questions; instances of no response have been excluded from the analysis below, leading to variations in the gross numbers of responses.

Observations of PTD decision-making took place for one working week in January 2020,^8^ in a busy, urban Magistrates’ Court (in fact, one of those observed in the original study). These were only conducted in publicly open hearings, relating to any type of adult criminal case – that is, those involving summary, either-way and indictable offences (all of which must start in Magistrates’ Courts). Since all cases should involve some form of PTD decision-making regardless of offence type, observations were not selected on this basis. To complement the survey, observations sought to objectively evaluate the impact of the amendments on day-to-day PTD practice. The rationale for observations as opposed to desk-based methods was the lack of available data on PTD practice from any other source. The process and practice of PTD hearings and the nature of decision-making in PTD hearings (for example, reasons, time taken, submissions by parties) are not routinely recorded and published. As such, it was necessary to directly observe proceedings in order to evaluate these aspects of practice. It is important to note that PTD decision-making in E&W does not generally have a dedicated hearing of its own (with the exception of bail appeals in the Crown Court); it forms only one part of all pre-trial criminal hearings. Therefore the observation of full hearings was necessary and involved matters irrelevant to the study, requiring a degree of judgement by the observer as to what aspect of a hearing represented ‘PTD decision-making’. It was also necessary to select hearings on a convenience basis, as PTD decision-making is not listed as a specific hearing type in advance. Hearings were selected on the basis that they were likely to involve PTD decision-making – generally, any hearing taking place prior to trial (with first appearances at court being almost certain to involve observable PTD decision-making). Where and when such hearings were occurring was determined by engaging with court staff, as there was a tendency to list such hearings for the same court room. For the purposes of this study (reflecting the 2016 study), PTD decision-making was defined exclusively as:

the period during an observed hearing in a single criminal case during which the court (which includes members of the judiciary; lawyers; the defendant(s); and any witnesses) engages with the issue of whether a defendant should be released on bail or remanded in custody (detained) until the next hearing.

All other matters observed during hearings were, generally, not recorded unless they provided valuable contextual information. A Hearing Monitoring Form was used to record information relevant to the objectives of the study, including basic relevant, case details (all of which was publicly accessible, since the hearings being targeted were open to the public) in order to identify each observation. The form recorded information about the submissions of the parties in hearings; the decision-making process of the court and outcome in relation to PTD; and made note, particularly, of the time spent on submissions and decision-making, and any issues which arose in relation to disclosure of evidence/information to the defence. A total of 28 hearings involving PTD decisions (as defined above) were observed across five days. Again, not all of the issues relevant to this study were observed in all hearings. The limitations of the study must be recognised in assessing its validity. The numbers of respondents and observations are small, and relate to short time periods. The observations occurred in only one location, did not cover PTD in the Crown Court, and were not subject to peer review. Additionally, the survey did not manage to capture the views of DJs and DDJs. However, notwithstanding these points, the data gathered does provide some useful insight into the effectiveness of these amendments in addressing problems with PTD practice, and (at present) represent the only source of information on this subject.

## Results and Discussion

The overarching aim of the fieldwork was to ascertain the impact of the amendments to the CrimPR on PTD practice. To determine this, analysis of the data explored two major themes: Awareness and Impact. The analysis below explores how aware respondents were of the amendments generally; what impact (if any) respondents perceived the amendments to have had; and what impact (if any) the amendments had had based on observations of court proceedings.

### Awareness of the Amendments

The survey data suggested generally high levels of awareness of all of the amendments, amongst both LMs and DPs, either at the time the amendments came into force (April 2017) or at some point afterwards. There was particularly high awareness of the amendments relating to the need for sufficient time to be provided for the defence to consider newly disclosed information or evidence (79% (26/33) of respondents were aware of this); and the requirement for detailed and specific reasoning in the announcement of PTD decisions (85% (28/33) of respondents were aware of this). Respondents were least aware of the requirement for the court to ensure sufficient time to consider the parties’ representations and to make their decision (61% (20/33) of respondents were aware of this). Whilst this represents a majority of respondents, it does suggest a significant proportion were not familiar with this obligation. This could be interpreted in a few ways. It might suggest simply that the relevant parties to PTD decision-making were aware that they should, as a matter of good practice, spend sufficient time on such matters but were not aware of the legal requirement to do so. Alternatively, it might infer that devoting more than a brief period of time to PTD decision-making was not a priority for decision-makers and practitioners. Considering previous findings regarding the brevity of proceedings (reflected in the observations – see below), the latter conclusion seems reasonable. A possible explanation for this is the need to manage a busy workload (significantly increased since the advent of the Covid-19 pandemic, though predating it) with limited resources. Alternatively, it may be that practitioners simply did not place high value on such decisions, regarding them as largely straightforward, “bread and butter” type work. Additionally, the vagueness of the term ‘sufficient time’, and consequent scope for wide interpretation, arguably enables practitioners to interpret their decision-making as always being compliant and therefore to disregard the requirement as lacking any practical meaning. As a result, some practitioners may have simply paid little heed to the concept of sufficient time in this context. Overall though, the data suggested high levels of awareness of the amendments, or at least the obligations introduced by them.

### Impact and Change in Practice

Notwithstanding the above, the data also suggested that the amendments had had little impact (defined for the purposes of this study as ‘some change in behaviour or practice by any practitioner(s) during the course of pre-trial detention (i.e. bail and custody) decision-making, as a result of the amendments’).

#### Providing ‘Sufficient Time’ for the Defence to Consider IDPC

In relation to the amendment requiring the provision of sufficient time to consider late disclosure by the prosecution, 33% (7/21) of respondents indicated there had been ‘no impact’; 29% (6/21) indicated ‘limited impact’; and 19% (4/21) indicated ‘some impact’. Some of the narrative responses provided by respondents supported the conclusion that the amendment had not been impactful or led to change. One DP stated, ‘I would not have allowed a case to proceed until I had all necessary information… as such the change had little impact’ (DP6); whilst one LM commented, ‘[i]n practice, the defence would have IDPC and time to consider before [a] bail app[lication], before the change’ (LM1). However, others felt that the granting of additional time was now even less likely, with one respondent stating that ‘adjournments are now routinely denied, and clerks will advise against them’ (DP5).^9^ The narrative responses suggested respondents had mixed views on whether this lack of impact was actually a problem. For example, one respondent argued that ‘[c]ourts are obsessed with dealing with cases as quickly as possible to get through the list… [and have] no real regard to this at all’ (DP3); whilst another felt that ‘the court generally doesn’t care about provisions that assist the defence.’ (DP2). However, some respondents indicated that they had either granted time to the defence for this purpose (three LMs); or been granted time (four DPs). One respondent commented that LMs ‘often give the defence time to consider information’ (LM6), whilst another stated that ‘I am usually given time but that’s only when court isn’t too busy’ (DP7). The observation data suggested a problem may exist (at least in a minority of cases), with a few examples of late disclosure observed during proceedings or IDPC being described by the court as incomplete. During one observation, the court granted additional time for the defence to consider late IDPC, suggesting compliance with the amendment – but this was exceptional.

#### Providing Disclosure

In relation to the amendment requiring that all material relevant to a PTD decision be disclosed to the defence as soon as is practicable, the data again suggested little or no impact from the perspective of the respondents, and therefore that there had been little or no change in practice. Of those respondents who answered the question on impact and were aware of the amendment, the majority thought it had had no (9/21, 43%) or limited (5/21, 24%) impact, with a small proportion believing it had had ‘some’ impact (4/21, 19%). None believed it had had ‘significant’ impact. 75% (15/20) of respondents thought that there had been no change in practice related to IDPC and disclosure. Narrative responses largely affirmed the ‘low or no impact’ conclusion. 21% (7/33) of respondents provided comments, only one of which inferred any impact had occurred (the comment suggested that this requirement could ‘occasionally’ be used to ensure that information material to PTD was provided). LMs did not perceive this to be a problem; in contrast, DPs clearly did, citing the following as common issues: a lack of information provided about cases; the large caseloads of the CPS; a general emphasis on speed in hearings; and a lack of enforcement when disclosure failures occurred. For example, one respondent commented that defence lawyers ‘are pressurised into getting their cases heard, and there is a lack of regard for sufficient time and disclosure’ (DP5); whilst another suggested impact would only occur if ‘court clerks robustly advised Magistrates to require it or if the prosecution was held to account by the Magistrates’ (DP7), implying that this was generally not the case in practice. Unusually, one LM commented that they had noticed ‘no change whatsoever’ and that ‘prosecutors provide little information’ (LM6), suggesting that some judicial figures (albeit a minority) may have recognised a persistent problem in this area.

However, other LMs regarded the amendment as redundant because the prosecution ‘had that obligation anyway’ (LM5), and therefore it was ‘difficult to see what purpose the rule changes serve’ (LM1). Indeed, one felt that ‘as far as I am aware the prosecution has always made full disclosure on elements affecting the bail decision before a bail decision is made’ (LM8), a quite contrasting view when compared with the DP responses. This might be explained by regional variations across E&W. For example, one respondent said: ‘courts in [Region One] are pretty organised. Not the same in [Region Two] where you barely receive papers’ (DP4). Alternatively, it might be explained by a disinclination of LMs to recognise a problem, as that would imply a failure on their part to ensure compliance with the rules. In observations, issues related to disclosure of information and evidence which were material to remand occurred in a small number of cases, and were primarily about incomplete pre-hearing disclosure or mid-hearing disclosure by the prosecution or requests by the defence. For example, in one hearing the defence had not received a victim personal statement as part of the IDPC;^10^ and unused material was accessed and examined by the defence as the hearing progressed.^11^ Overall, data suggested that this amendment had had little or no impact from the perspective of respondents, and therefore there had been little or no change in practice. The 2016 study identified ‘the unsatisfactory position regarding disclosure’, which was often late, minimal or incomplete (Cape & Smith, 2016); as such, the follow-up study suggests such issues persist, though LMs did not appear to recognise this. A lack of impact might imply that disclosure of relevant material is, indeed, generally not an issue; or underline a continuing problem which has not been addressed by the amendment. The observation data might suggest that a problem does exist (at least in a minority of cases). Considering that this amendment was intended to benefit defendants (and by extension, DPs) by ensuring adequate disclosure, the fact that DPs tended to be critical of its lack of impact suggests no such benefit has materialised. In summary, the amendment has failed to address the issue because it has not translated into changed practice.

#### Ensuring ‘Sufficient Time’ for PTD Hearings and Decisions

As with the other amendments, the introduction of a requirement for courts to ensure sufficient time is spent on PTD hearings appeared to have had little impact. 70% (14/20) of respondents thought that this amendment had had ‘limited’ or ‘no’ impact, with 75% (15/20) indicating there had been no change in the amount of time spent on PTD decision-making; only 15% (3/20) believed it had had some impact, whilst 15% (3/20) were not aware of this amendment and the consequent requirement. These findings might be explained by the belief amongst respondents that sufficient time was generally made available for PTD hearings and that this had always been the case. For example, one respondent felt that ‘courts have always taken time to consider submissions and its decision on bail’ (LM1), whilst another stated, ‘I have never felt rushed to reach a decision regarding bail’ (LM7). Indeed, when directly asked, 85% (17/20) of respondents (the majority being DPs) stated that courts had sufficient time for such decisions. Various comments (mostly, but not exclusively, from LMs) argued that they took PTD seriously; were not rushed; and did not feel pressured to curtail decisions due to a lack of time. However, several narrative responses suggested the opposite, with respondents variously arguing that ‘courts routinely rush through decisions’ (DP7); that ‘there is always time pressure in Magistrates’ Court[s]’ (LM6); that there were ‘too many cases listed in one court, not enough time’ (DP7); and that ‘due to closure of many courts [PTD cases] are now in one overrun court, so less time is available’ (DP7). An interesting point was made by one LM, arguing that even if LMs (as decision-makers) were ‘prepared to take sufficient time to consider everything’, workload pressures on parties might prevent them from doing do, thus ‘the lawyers don’t present us much information’ (LM6). This therefore draws a distinction between different causes of insufficient time being spent on PTD decisions: on the one hand, as a result of practical limitations on time and resources; and on the other, due to a general unwillingness or antipathy to spending time on such matters.

Observation data regarding time spent on PTD hearings reflected previous findings. In 26 cases, the overall length of the hearing and the time spent exclusively on PTD matters were recorded. 38% (10/26) of hearings lasted between 10 and 20 min; with 35% (9/26) lasting 5 to 10 min. A very small number lasted under 5 min, or over 20 min. As such, most observed hearings fell within the 5 to 20 min range. Between 1 and 5 min was spent on PTD matters in 65% (17/26) of hearings, with just under 20% (5/26) spending 5 to 10 min on such matters. In terms of the proportion of a hearing spent considering PTD matters, nearly 30% (7/26) of hearings spent less than 10% of the overall time to consider PTD; whilst just over 40% (11/26) spent under a third of the overall time on PTD matters. This initially suggests that a fairly small proportion of each hearing was devoted to PTD decision-making, with fairly limited amounts of time (a matter of minutes in most cases) being spent on such matters. This could of course be entirely appropriate, depending on the overall length of the hearing; the general nature of the case; the positions of the party in relation to detention or bail; and the complexity of the PTD decision at hand.

As a general comment, it might be highlighted that most of the cases recorded in this manner were first appearances at court,^12^ involving (primarily) summaries of cases, entering of a plea, case management (such as setting dates), as well as PTD matters. Considering that PTD matters potentially involve deprivation of liberty (entailing, arguably, the consideration of a range of important issues), these figures suggest ‘sufficient time’ is not spent on such matters. Indeed, in 50% (5/10) of hearings in which the defendant was detained, discussion of PTD represented less than a third of overall time; and in 60% (6/10) of hearings involving a detention, less than 5 min was spent on such matters. One aspect that might be expected to involve a significant amount of time are the representations of the parties – for example, the CPS opposing the default right to bail, and the defence arguing for release (perhaps with conditions). Yet, in nearly 70% (18/26) of hearings, the CPS either made no representations relating to PTD or made representations lasting less than 1 min. Defence lawyers spent only slightly more time, with 65% (17/26) either making no representations or advocating on PTD matters for under a minute. In only two cases were representations longer than 5 min. This reflects findings from the 2016 study, which found that many hearings were essentially uncontested, with little adversarial debate about PTD (Cape & Smith, 2016). One aspect that could not be adequately measured was decision-making by LMs, as they often retired to consider decisions. Whilst this length of time *could* have been measured, there was no certainty as to what matters were being discussed and this would therefore have been an unreliable metric.

#### Reasoning for PTD Decisions

As above, the amendment requiring the announcement of decisions related to PTD to be linked to the specific features of a case appeared to have limited impact on practice. 75% (15/20) of respondents felt there had been ‘limited’ or ‘no’ impact, with 20% (4/20) believing there had been ‘some’ impact. Narrative responses reflected this, but were mixed in terms of whether the lack of impact was a problem. Some stated that they had always ‘announced’ (LM1) decisions or been ‘required to give reasons’ (LM7). This does however suggest a misunderstanding of the amendment, which relates to the *nature* of the announcement of decisions – not their mere existence. These answers did not seem to recognise the difference between announcing/giving reasons, and giving detailed and specific reasons (the primary thrust of the amendment). Concern about this was reflected in several narrative answers, with respondents suggesting that ‘short reasons are often given’ (DP3); that reasoning often lacked ‘clarity or logic’ (DP5); that ‘[M]agistrates can simply pay lip service to the grounds for remand without properly applying them’ (DP5); and that ‘fairly generic language is still used’ (LM6).

Observation data seemed to confirm these concerns. Reasoning for PTD decisions was provided in less than half of the 27 observed hearings in which this was recorded – with nearly 60% (16/27) providing no reasons for the PTD decision. Where reasons were announced by the court, a key measure of compliance with the amendment was the specificity of the announcement. For the purposes of the study, a specific announcement was defined as:

The provision of reasoning or explanation which mentioned the grounds for refusing bail or imposing conditions; cited factors for such grounds being established (for example, linking a fear of further offences to actual behaviour by the defendant); or explicitly relied on specific facts or circumstances in a case, or the arguments presented by lawyers.

Of those cases that involved some form of announcement of a PTD decision, 45% (5/11) had specific reasons given – accounting for less than 20% of all cases observed. Troublingly, of those cases where reasoning had none or few of the features mentioned above, more than 80% (5/6) involved the refusal of bail and therefore the detention of a defendant. This is concerning considering the significant impact detention could have on future decisions about further detention (as well as the impact of detention itself on personal wellbeing, employment, family, and housing).^13^ Without clear and detailed reasoning, a defendant might perceive the decision to be arbitrary, unjust or without basis; furthermore, the defendant is hindered in their ability to challenge continuing detention at future hearings because they cannot scrutinise and question the specific rationale for the decision. Finally, the actual announcement of decisions was brief, with over half lasting less than one minute. Together, the data on reasoning therefore paints a picture of hearings which regularly lacked any reasoning; or reasoning which was brief, generic and lacking detail (reflecting previous findings). This therefore suggests a lack of compliance with the amendment, and by extension implying the amendment has not been impactful.

## Conclusions

Despite evidence that respondents were aware regulatory changes had occurred in relation to PTD decision-making (as regards disclosure, sufficiency of time, and announcement of decisions), they appeared to believe that very little had changed in their day-to-day work. This was, generally, confirmed by observation data, particularly in relation to sufficiency of time and announcement of decisions. Importantly, whilst the consensus appeared to be that the amendments had had little impact, there were mixed views on whether or not this mattered. LMs tended to perceive a lack of impact as unproblematic – either because the amendments affirmed what they considered to be their pre-existing practice or because they did not consider a problem to exist, rendering the amendments unnecessary. In contrast, most DPs tended to consider the lack of impact as important because it meant pre-existing issues remained unaddressed, and suggested that regulatory changes designed to improve practice were either ignored or not enforced consistently. As such, there was a clear distinction between the views of the judiciary and lawyers. Observation data regarding the length of time spent on PTD hearings and the announcement of PTD decisions appeared to broadly favour the negative conclusions of DPs. Representations, announcements and decision-making were generally characterised by brevity; whilst this is sometimes appropriate (for example, if a prosecutor did not oppose unconditional bail), it is arguably not in cases involving the removal of liberty. Announcements were frequently lacking clarity or detail, or simply did not explicitly state why a decision had been made (and in several cases did not state the decision at all). Compliance with the amendment requiring some reference to the specifics of a case was rare. Whilst it might be argued that grounds and reasons could be inferred from the proceedings leading up to the announcement, it should be noted that this does not reflect the law and assumes that all persons present (including defendants and witnesses) can fully comprehend what is happening and why. For example, one might question whether an unrepresented defendant (several were observed during the study) would be able to understand the reasons for detention without a clear announcement, and therefore might be unable to effectively challenge it (or might simply perceive it as unfair).^14^ As such, whilst some practitioners did not regard the lack of impact of the amendments as problematic, the observation data might suggest otherwise, certainly in regards to announcements and the sufficiency of time spent.

Overall, the data therefore broadly suggests that the amendments have not had any meaningful impact and that the problems identified in previous research persist. In short, whilst the law in relation to PTD may have changed, this study suggests that cultural practices in relation to this type of decision-making have not. In the context of E&W, this raises questions about the utility of affecting meaningful change in practice through independent academic research and legal reform at a policy-making level. Whilst research in this area did catalyse legal reform, arguably, the more important question is whether this subsequently led to practical, long-term change in PTD practice. The findings of the follow-up study described in this article suggest little had changed, indicating a failure to transform legal reform into practical change. One potential explanation for this is that amending legal rules in this manner is both ‘top-down’ and static; such change makes demands of practitioners at a policy level without engaging in a sustainable programme of culture change at the “coal face” of practice. For example, this might be achieved by engaging directly with practitioners (particularly prosecutors and the judiciary) as well as defence practitioners, through focused training on such matters or ongoing consultation about the issues in question. It was beyond the remit of this study to examine training,^15^ but the lack of change in practice suggests that training (if any is provided) on the legal requirements related to PTD is currently ineffective in ensuring that practice reflects the law. Arguably, the legal tools now exist to address some of the issues affecting PTD practice in E&W, which was an important and significant step forward. However, influencing the awareness, motivation and behaviour of practitioners to better reflect these legal requirements on a daily basis appears to be more important to achieving a real change in practice. This study suggests a gap between law and practice, which needs to be addressed going forward. Current evidence on PTD in E&W suggests that the numbers of defendants detained prior to trial are approaching record highs; that defendants are being detained for longer periods, possibly unlawfully (Fair Trials, 2021b); and that prison conditions for those on remand are poor (Fair Trials, 2021a). In this context, it is more important than ever to ensure that PTD decision-making reflects the thorough, substantiated and well-reasoned ideal envisioned by the legal framework, so that detention is reserved for use in only those cases which truly merit it.

## Data Availability

The data described in this article is not currently publicly available.

## Footnotes

1 Defendants can – and increasingly do – attend PTD hearings ‘virtually’: that is, they will observe and interact with proceedings through live streaming technology. This is commonly used for defendants who are in custody at the time of a hearing. For defendants (in attendance or not) who do not have a lawyer, they may be able to use a ‘duty’ lawyer: a defence representative made available, free of charge, to defendants who do not have a legal representative of their own for a hearing, working on a rota basis.

2 The definition of ‘developed nations’ is taken from the United Nations’ World Economic Situations and Prospects (WESP) country classification (United Nations 2020).

3 Lay magistrates are part-time, unpaid volunteers who exercise judicial powers in magistrates courts. Whilst they must satisfy certain criteria and undertake training, they do not need legal qualifications. They hear the vast majority of criminal cases in E&W.

4 The CPS are the independent agency responsible for prosecuting most criminal cases in E&W.

5 It is important to note that the survey opened shortly before the UK’s nationwide Covid-19 lockdown, and remained open for the first few months of the initial lockdown period. Whilst the survey was available online, it is unclear what impact the pandemic may have had on the willingness of practitioners to engage with it, considering the unprecedented professional and personal challenges they would have faced at that time.

6 The survey was only targeted at practitioners who operate in magistrates’ courts, as the CrimPR 2020 only strictly apply to cases heard in such courts. For this reason, Crown Court judges and barristers were not part of the target sample.

7 Whilst the latter category of respondents were not asked to specify their professional status further, they were likely to be solicitors who most commonly provide defence representation in Magistrates’ Courts.

8 Five full court days – normally, 10am to 4pm. This was prior to any impact as a result of Covid-19.

9 A Clerk, or Legal Adviser as they are formally titled, is a qualified lawyer who advises LMs on the relevant law during hearings.

10 A victim personal statement is a written or oral statement in court which gives victims the opportunity to express how an offence has impacted on them. This is only relevant to sentencing, and does not form part of a case against a defendant.

11 Unused material is information or evidence gathered in the investigation of a crime but which the prosecution are not using as evidence in their case against a defendant. Unused material can contain information of value to the defence, and therefore they may wish to access this.

12 That is, the first appearance by a defendant after being charged with an offence.

13 Previous evidence suggests that those who are detained are more likely to be detained in future hearings (Cape & Smith, 2016; Transform Justice, 2018).

14 It might be noted that all defendants in detention (or facing the prospect of it) should be legally represented when such matters are being considered by a court, such is the significance and likely impact of the decision being made.

15 Though this might represent a future path for research in this area.

## Code Availability

N/A.
